# Pre-hospital rule-out of non-ST-segment elevation acute coronary syndrome by a single troponin: final one-year outcomes of the ARTICA randomised trial

**DOI:** 10.1093/ehjqcco/qcae004

**Published:** 2024-01-17

**Authors:** Goaris W A Aarts, Cyril Camaro, Eddy M M Adang, Laura Rodwell, Roger van Hout, Gijs Brok, Anouk Hoare, Frank de Pooter, Walter de Wit, Gilbert E Cramer, Roland R J van Kimmenade, Eva Ouwendijk, Martijn H Rutten, Erwin Zegers, Robert-Jan M van Geuns, Marc E R Gomes, Peter Damman, Niels van Royen

**Affiliations:** Department of Cardiology, Radboud University Medical Centre, Nijmegen, The Netherlands; Department of Cardiology, Radboud University Medical Centre, Nijmegen, The Netherlands; Department of Health Evidence, Radboud Institute for Health Sciences, Nijmegen, The Netherlands; Department of Health Evidence, Radboud Institute for Health Sciences, Nijmegen, The Netherlands; Ambulance Service, Safety Region Gelderland-Zuid, Nijmegen, The Netherlands; Ambulance Service, Safety Region Gelderland-Zuid, Nijmegen, The Netherlands; Ambulance Service, Witte Kruis, Houten, The Netherlands; Ambulance Service, Witte Kruis, Safety Region Noord-en Oost-Gelderland, Elburg, The Netherlands; Ambulance Service, Witte Kruis, Safety Region Zeeland, Goes, The Netherlands; Department of Cardiology, Radboud University Medical Centre, Nijmegen, The Netherlands; Department of Cardiology, Radboud University Medical Centre, Nijmegen, The Netherlands; General Practitioner Centre Nijmegen and Boxmeer, Nijmegen, The Netherlands; General Practitioner Cooperative Noord-Limburg, Venlo, The Netherlands; Department of Cardiology, Canisius Wilhelmina Hospital, Nijmegen, The Netherlands; Department of Cardiology, Radboud University Medical Centre, Nijmegen, The Netherlands; Department of Cardiology, Canisius Wilhelmina Hospital, Nijmegen, The Netherlands; Department of Cardiology, Radboud University Medical Centre, Nijmegen, The Netherlands; Department of Cardiology, Radboud University Medical Centre, Nijmegen, The Netherlands

**Keywords:** Acute coronary syndrome, Point-of-care troponin, Ambulance, Healthcare economics, Cost-effectiveness

## Abstract

**Background and aims:**

The healthcare burden of acute chest pain is enormous. In the randomized ARTICA trial, we showed that pre-hospital identification of low-risk patients and rule-out of non-ST-segment elevation acute coronary syndrome (NSTE-ACS) with point-of-care (POC) troponin measurement reduces 30-day healthcare costs with low major adverse cardiac events (MACE) incidence. Here we present the final 1-year results of the ARTICA trial.

**Methods:**

Low-risk patients with suspected NSTE-ACS were randomized to pre-hospital rule-out with POC troponin measurement or emergency department (ED) transfer. Primary 1-year outcome was healthcare costs. Secondary outcomes were safety, quality of life (QoL), and cost-effectiveness. Safety was defined as a 1-year MACE consisting of ACS, unplanned revascularization, or all-cause death. QoL was measured with EuroQol-5D-5L questionnaires. Cost-effectiveness was defined as 1-year healthcare costs difference per QoL difference.

**Results:**

Follow-up was completed for all 863 patients. Healthcare costs were significantly lower in the pre-hospital strategy (€1932 ± €2784 vs. €2649 ± €2750), mean difference €717 [95% confidence interval (CI) €347 to €1087; *P* < 0.001]. In the total population, the 1-year MACE rate was comparable between groups [5.1% (22/434) in the pre-hospital strategy vs. 4.2% (18/429) in the ED strategy; *P* = 0.54]. In the ruled-out ACS population, 1-year MACE remained low [1.7% (7/419) vs. 1.4% (6/417)], risk difference 0.2% (95% CI −1.4% to 1.9%; *P* = 0.79). QoL showed no significant difference between strategies.

**Conclusions:**

Pre-hospital rule-out of NSTE-ACS with POC troponin testing in low-risk patients is cost-effective, as expressed by a sustainable healthcare cost reduction and no significant effect on QoL. One-year MACE remained low for both strategies.

## Graphical Abstract

**Graphical Abstract:**
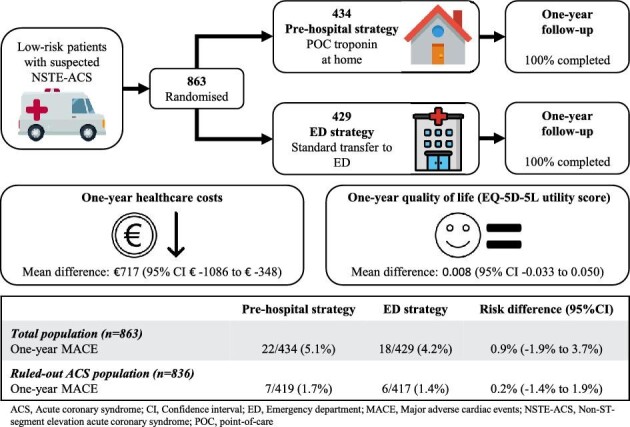
Overview of the study design and outcomes.

Key learning points **What is already known**

- Pre-hospital rule-out of non-ST-segment elevation acute coronary syndrome (NSTE-ACS) with point-of-care (POC) troponin measurement in low-risk patients reduces 30-day healthcare costs.
- The incidence of major adverse cardiac events (MACE) at 30 days after pre-hospital rule-out of NSTE-ACS in low-risk patients is low and comparable to the MACE incidence after standard transport to the emergency department (ED).

**What this study adds**

- Pre-hospital rule-out of NSTE-ACS in low-risk patients is cost-effective at 1 year, as expressed by a sustainable healthcare cost reduction and no difference in quality of life.
- At 1 year, the safety of the pre-hospital rule-out of NSTE-ACS was comparable to the safety of standard transport to the ED.

## Introduction

Chest pain is the most common reason for emergency medical contact.^1^ Due to a lack of validated pre-hospital algorithms to rule out non-ST-segment elevation acute coronary syndrome (NSTE-ACS), patients with chest pain are routinely transported to the emergency department (ED).^2,3^ Around 10% of all ED visits are due to chest pain, which contributes to ED overcrowding, a growing problem worldwide that is associated with high costs, increased length of stay, and worse clinical outcomes.^4,5^ Importantly, for the majority (80–90%) of patients suspected of NSTE-ACS, an acute coronary syndrome (ACS) can be ruled out.^6–8^ Furthermore, low-risk patients are not likely to benefit from expensive, lengthy, and resource-intensive ED visits.^1,9,10^ The HEART [History, Electrocardiogram (ECG), Age, Risk factors, and troponin] score enables rapid identification of low-risk patients with low short-term major adverse cardiac event (MACE) incidence.^11,12^ A point-of-care (POC) troponin measurement enables pre-hospital identification of low-risk patients by ambulance paramedics.^13^ Recently, in the ‘Acute rule-out of non-ST-segment elevation ACS in the (pre)hospital setting by HEART score assessment and a single point-of-care troponin’ (ARTICA) trial, we found that pre-hospital rule-out of NSTE-ACS in low-risk patients led to a large healthcare cost reduction in the first 30 days, while the occurrence of MACE was very low. However, the long-term healthcare costs, impact on safety, quality of life (QoL), and cost-effectiveness of the pre-hospital rule-out of NSTE-ACS are unknown. Here we present the final 1-year results of the ARTICA trial.

## Methods

### Trial design

The ARTICA trial is an investigator-initiated, multicentre, open-label, randomized controlled trial. The study design and the primary 30-day results have been published previously.^14,15^ An independent and blinded Clinical Events Committee (CEC) was responsible for the adjudication of the MACE, and a Data and Safety Monitoring Board (DSMB) oversaw the safety, conduction, and progress of the trial. The study was conducted in accordance with the principles of the Declaration of Helsinki, the Medical Research Involving Human Subjects Act (WMO), and the statements of the Dutch Central Committee on Research Involving Human Subjects (CCMO). The medical ethics committee in Oost-Nederland, The Netherlands, approved the trial on 27 November 2018 (NL66755.091.18). The trial was registered at Clinicaltrials.gov (NCT05466591).

### Participants

The study population consisted of low-risk patients suspected of having a NSTE-ACS who had an onset of symptoms ≥2 h before ambulance arrival. Low-risk was defined as a HEAR score (HEART score without the troponin component) of ≤3 (Supplementary material online, *Figure S1*). The ambulance paramedics performed screening, the informed consent procedure, randomization, and the POC troponin measurement.

Patients were not eligible for participation if they were suspected of another diagnosis requiring ED presentation [e.g. aortic dissection or pulmonary embolism (PE)] or if they were unable to provide written informed consent. Supplementary material online, *Table S1* in the Supplementary Appendix depicts the complete list of inclusion and exclusion criteria.

### Randomization and intervention

Eligible patients were randomized to the pre-hospital rule-out strategy or the ED rule-out strategy. In the pre-hospital rule-out strategy, patients underwent POC troponin T measurement on-site. If POC troponin T was low (<40 ng/L), the care for the patient was transferred to the general practitioner (GP), as is the normal procedure for not transporting patients. Patients with elevated POC troponin T were transported to the ED. In the ED rule-out strategy, patients were directly transported to the ED without POC troponin measurement, according to standard practice. At the EDs in the Netherlands, the European Society of Cardiology 0h/1h algorithm is standard practice. POC troponin T was measured using the Cobas h232 (Roche Diagnostics, Basel, Switzerland). The detection limits are 40–2000 ng/L. Concentrations of 40–2000 ng/L on this assay are comparable to laboratory high-sensitivity troponin T concentrations.^16^

### One-year follow-up

Follow-up was performed by telephone and e-mail. If patients visited a hospital, medical records were collected to record all data on procedures and events. In case of non-response by a patient, the GP and hospital were contacted. Data were collected on all utilized healthcare resources over the entire period of evaluation (1 year). All patients received quality-of-life questionnaires.

### Healthcare resources

The prices for the healthcare resources were determined in accordance with the 2018 reference list of the Dutch National Healthcare Institute.^17^ The costs for hospital procedures were based on the standard 2018 list prices and the diagnosis-treatment combination (reimbursement system in the Netherlands).^18^ The costs for the index ambulance visit were considered equal in both strategies. Supplementary material online, * Table S2* shows a detailed description of the average prices used for analyses.

### Primary outcome

The primary outcome was healthcare costs at 1 year. Healthcare costs at 1 year consisted of the sum of all costs related to healthcare consumption, namely costs for ambulance transports, GP visits, hospital visits, additional tests, newly prescribed medications, and inpatient hospitalizations.

### Secondary outcomes

Secondary outcomes were safety, QoL, and cost-effectiveness. Safety was assessed by the incidence of MACE at 1 year, which was defined as one or more of the following events: ACS, unplanned revascularization, and all-cause death. Incidence of MACE at 1 year was compared between groups in the total population and in the ruled-out ACS population (all patients for whom an ACS was ruled out, either in the pre-hospital setting or in the ED). Quality of life was assessed at 30 days and 1 year and measured with a validated health-related QoL instrument, the EuroQol-5D-5L (EQ-5D-5L), for which a Dutch version is available.^19^ The EQ-5D-5L comprises five domains: mobility, self-care, usual activities, pain/discomfort, and anxiety/depression. The EQ-5D-5L utility was obtained by applying a predetermined weight to these five domains. Utility gives a global quantification of the patient's health status on a scale ranging from 0 (death) to 1 (perfect health).

Cost-effectiveness adheres to an incremental approach and was defined as the difference in healthcare costs divided by the difference in EQ-5D-5L utility scores at 1 year.

### Statistical analysis

The statistical analysis plan for the entire study was published previously.^15^ Categorical data were summarized by (relative) frequencies and percentages and compared using Fisher's exact test or the chi-squared test, whichever was most appropriate. Continuous data were summarized as means ± SD or medians [interquartile ranges (IQRs)]. Healthcare costs were expressed as means ± SD. Mean cost differences and their 95% confidence intervals (CIs) were calculated. To take a potentially skewed distribution and possible heteroscedasticity into account, a generalized linear model (GLM) with a gamma distribution for the dependent variable was included. For the MACE endpoints, 1-year incidence was calculated in both groups in the total population and in the ruled-out ACS population. Point estimates for the risk differences were calculated along with their 95% CIs. For the MACE incidence in the total population, a cumulative incidence curve was plotted, and time to event was compared between strategies using the log-rank test. For the MACE incidence at 1 year, sensitivity, specificity, positive predictive value (PPV), and negative predictive value (NPV) were calculated for both strategies, along with their 95% CIs (if possible).

The EQ-5D-5L utility scores were analysed using tobit regression with right-censoring at 1.

For the EQ-5D-5L utility scores, two missing data scenarios were performed: a scenario with complete cases and a scenario with multiple imputation by chained equations with predictive mean matching. Missing data were assumed to be missing at random. Cost-effectiveness was presented as the difference in costs between both strategies compared to the difference in their effect (EQ-5D-5L utility score at 1 year). Cost-effectiveness incorporating parameter uncertainty using bootstrapping was presented as a cost-effectiveness plan (CE-plane). With the ED rule-out strategy occupying the origin of the graph, the CE plane plotted the incremental costs (*y*-axis) and utilities (*x*-axis) of the pre-hospital rule-out strategy relative to this standard care in a two-dimensional (costs and utility scores) space. In the CE plane, the area above the horizontal axis represents an increase in costs, and the area to the right of the vertical axis represents a gain in QoL. There are four quadrants. In two quadrants, there is an Incremental Cost Effectiveness Ratio (ICER) (upper right-hand quadrant and lower left-hand quadrant), in the other two quadrants there is no interpretable ICER as there is no trade-off between costs and effects. The CE plane, incorporating uncertainty, shows the joint distribution of differences in costs and utility scores at 1 year recalculated over 1000 replications of the imputed study data using the bootstrap resampling method. Each point in the scatterplot represents one bootstrap sample. The scatter cloud may lie in more than one quadrant. From the CE-plane a Cost-Effectiveness Acceptability Curve (CEAC) could be inferred, which shows the likelihood that the pre-hospital rule-out strategy is cost-effective across a range of cost-effectiveness thresholds (willingness to pay for an effect). Data analyses were performed using Stata version 17 (StataCorp LLC, College Station, TX, USA) and SPSS statistics version 27 (IBM, Armonk, NY, USA).

## Results

### Participants

**Figure 1:**
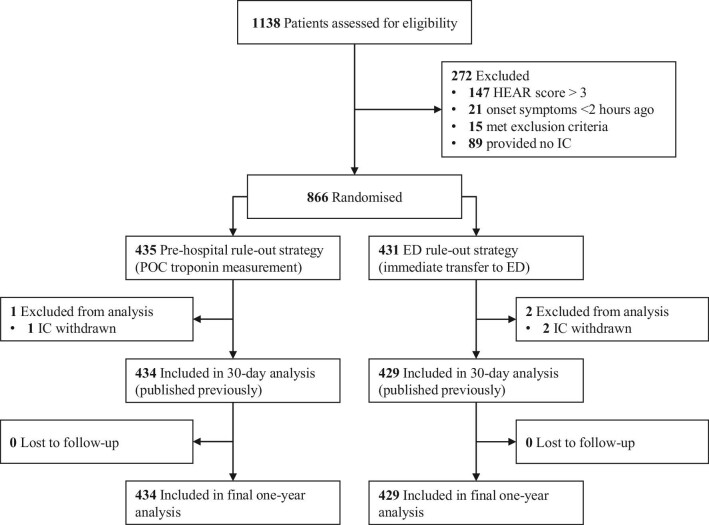
Study flow chart.

From 1 March 2019 to 4 May 2022, 1138 patients were screened, and a total of 866 patients were randomized. Three patients were excluded from the analyses after withdrawal of informed consent (*n* = 3). One-year follow-up was completed for all patients, resulting in a total of 863 patients available for analysis. The study flow chart is depicted in *Figure 1*. The patients had a mean age of 54 ± 13 years, a median HEAR score of 3 (IQR 2–3), and 57% of the study population was female. In the pre-hospital rule-out strategy, 37/434 patients (8.5%) were transported to the ED because of elevated POC troponin T (*n* = 18), failed POC troponin T test (*n* = 12), or decision of the GP (*n* = 7). In the ED rule-out strategy, 100% of the patients were transported to the ED. In 836 patients (96.9% of the total population), ACS was ruled out, either in the pre-hospital setting or at the ED. *Table 1* shows the baseline characteristics.

**Table 1:** Baseline characteristics

Variable	Pre-hospital rule-out strategy *N* = 434	ED rule-out strategy *N* = 429
Age (years), mean ± SD	53.7 (13.1)	53.2 (12.5)
Female sex *N* (%)	247 (56.9)	248 (57.8)
HEAR score, median (IQR)	3 (2–3)	3 (2–3)
History score,^a^ median (IQR)	0 (0–1)	1 (0–1)
ECG score,^a^ median (IQR)	0 (0–0)	0 (0–0)
History of atherosclerotic disease *N* (%)	34 (7.8)	23 (5.4)
Hypertension *N* (%)	85 (19.6)	71 (16.6)
Diabetes mellitus *N* (%)	22 (5.1)	14 (3.3)
Current smoker *N* (%)	112 (25.8)	110 (25.6)
Hypercholesterolaemia *N* (%)	37 (8.5)	35 (8.2)
Family history positive *N* (%)	155 (35.7)	152 (35.4)
BMI ≥30 kg/m^2^*N* (%)	86 (19.8)	80 (18.6)
Onset of symptoms >24 h *N* (%)	130 (30.0)	123 (28.7)
Onset of symptoms <24 h *N* (%)	304 (70.0)	306 (71.3)
Onset (h) of symptoms (if onset < 24 h), mean ± SD	7.1 (5.2)	6.8 (5.3)
Elevated troponin *N* (%)	18 (4.1)	24 (5.6)
Heart rate (bpm), mean ± SD	77.9 (13.2)	77.4 (13.4)
Systolic blood pressure (mmHg), mean ± SD	146.0 (20.5)	145.1 (21.2)

BMI, body mass index; Bpm, beats per minute; ECG, electrocardiogram; ED, emergency department; h, hours; HEAR score, History, ECG, Age and Risk factors score; IQR, interquartile range; SD, standard deviation.

a According to the HEAR score.

### MACE in the total population

**Figure 2:**
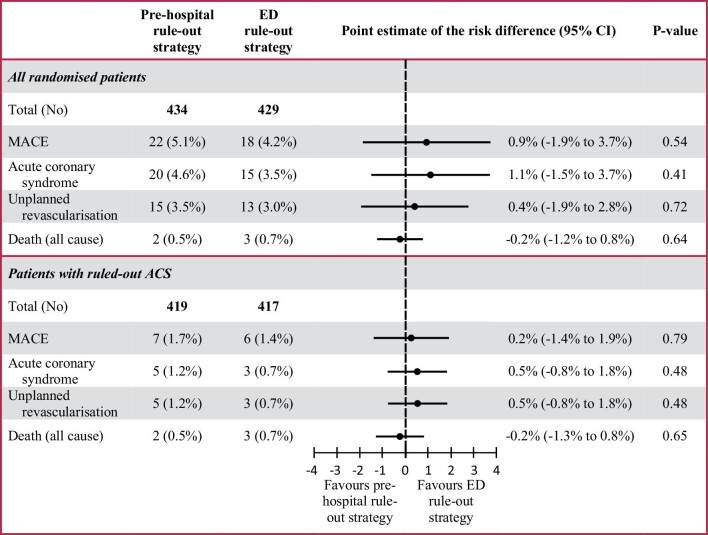
Incidence of major adverse cardiac events at 1 year.

In the total population, MACE at 1 year (including MACE at index presentation) occurred in 22/434 patients (5.1%) in the pre-hospital rule-out strategy vs. 18/429 patients (4.2%) in the ED rule-out strategy, with a point estimate for the risk difference of 0.9% (95% CI −1.9% to 3.7%, *P* = 0.54) (*Figure 2*). The pre-hospital rule-out strategy had a sensitivity of 68.2% (95% CI 47.5% to 84.9%), specificity of 100%, PPV of 100%, and NPV of 98.3% (95% CI 96.8% to 99.3%) for MACE at 1 year. In the pre-hospital rule-out strategy, 20 patients (4.6%) had an ACS, of which 14 patients (3.2%) had a non-ST-segment elevation myocardial infarction (NSTEMI), 5 patients (1.2%) had an ST-segment elevation myocardial infarction (STEMI), and 1 patient (0.2%) had unstable angina. Unplanned revascularization was performed in 15 patients (3.5%), and 2 patients (0.5%) died. A total of 15 of the 22 patients were directly transported to the ED because of either an elevated POC troponin T concentration (*n* = 14) or a failed test (*n* = 1).

The ED rule-out strategy had a sensitivity of 66.7% (95% CI 43.7–85.2%), a specificity of 100%, a PPV of 100%, and an NPV of 98.6% (95% CI 97.1–99.4%). In the ED rule-out strategy, 15 patients (3.5%) had an ACS, of which 11 patients (2.6%) had an NSTEMI and 4 patients (0.9%) had unstable angina. Unplanned revascularization was performed in 13 patients (3.0%), and 3 patients (0.7%) died.

**Figure 3:**
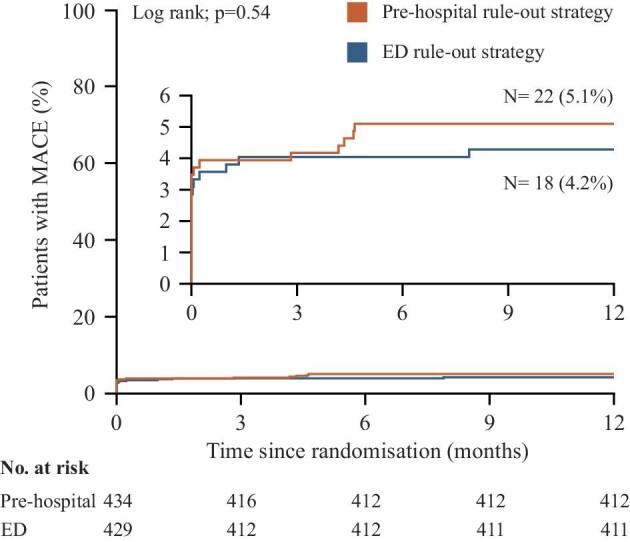
Cumulative incidence curve for the incidence of major adverse cardiac events at 1 year.

*Figure 3* shows a cumulative incidence curve for the incidence of MACE in both strategies in the total study population (*P* = 0.54).

### Safety pre-hospital rule-out of acute coronary syndrome

In the ruled out ACS population (96.9% of the total population), MACE at 1 year occurred in 7/419 patients (1.7%) in the pre-hospital rule-out strategy vs. 6/417 patients (1.4%) in the ED rule-out strategy, with a point estimate for the risk difference of 0.2% (95% CI −1.4% to 1.9%, *P* = 0.79).

In the pre-hospital rule-out strategy, 5 patients (1.2%) had an ACS, of which 2 patients (0.5%) had a NSTEMI, 2 patients (0.5%) had a STEMI and 1 patient (0.2%) had unstable angina. Unplanned revascularization was performed in 5 patients (1.2%), and 2 patients (0.5%) died. MACE after the first 30 days occurred in 5 patients (1.2%) in the pre-hospital rule-out strategy; 3 patients had an ACS (0.7%), and 2 patients (0.5%) died. One patient had a NSTEMI (0.2%) after 141 days, 1 patient had a STEMI (0.2%) after 127 days, and 1 patient had unstable angina (0.2%) after 132 days. All three of these patients presented with new symptoms and received revascularization. One patient died from end-stage myelodysplastic syndrome. One patient was found in a confused state and died while being transferred into the ambulance; no autopsy was performed.

In the ED rule-out strategy, 3 patients (0.7%) had an ACS, of which 3 patients (0.7%) had unstable angina. Unplanned revascularization was performed in 3 patients (0.7%), and 3 patients (0.7%) died. MACE after the first 30 days occurred in 2 patients (0.5%) in the ED rule-out strategy; 0 patients (0.0%) had an ACS, and 2 patients (0.5%) died. One patient died from thyroid cancer, and one patient committed suicide.

A list with details about all patients with MACE is available in the Supplementary Appendix (Supplementary material online, * Table S3*).

### One-year healthcare costs

**Figure 4:**
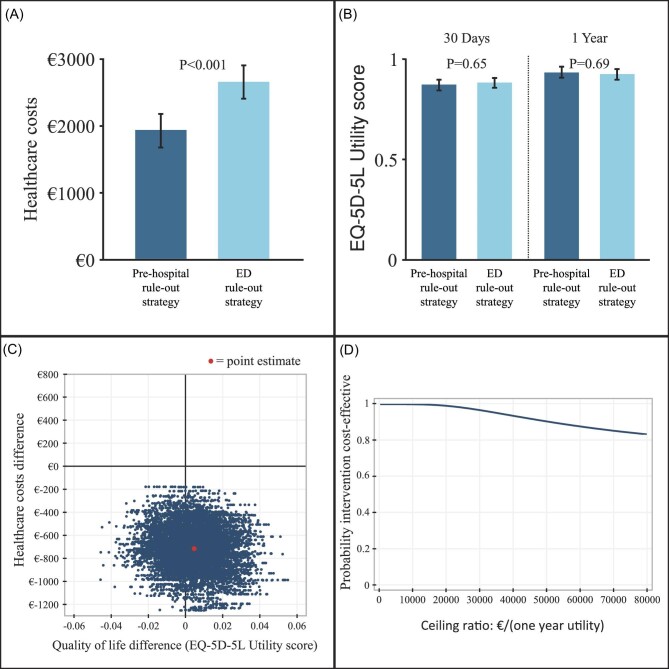
Mean healthcare costs (A), EQ-5D-5L utility scores (B), CE plane (C) and CEAC (D).

Healthcare costs were significantly lower in the pre-hospital rule-out strategy (€1932 ± €2784 vs. €2649 ± €2750), with a mean difference of €717 (95% CI €347–1087, *P* < 0.001). *Table 2* shows the healthcare costs and their components. *Table 3* shows the healthcare resource use. *Figure 4*A shows the healthcare costs.

**Table 2:** Mean healthcare costs and its components

Costs at 1 year (€)	Pre-hospital rule-out strategy *N* = 434	ED rule-out strategy *N* = 429	Mean difference (95% CI)	*P*-value
Healthcare costs	1931.83 (2784.01)	2648.63 (2749.56)	716.80 (347.07–1086.51)	<0.001
Ambulance transports	846.00 (291.07)	831.94 (319.67)	−14.06 (−54.90 to 26.78)	0.50
GP visits	52.09 (33.11)	25.00 (38.55)	−27.09 (−31.89 to −22.29)	<0.001
Hospital visits	603.94 (1530.36)	1314.17 (1450.37)	710.23 (510.97–909.48)	<0.001
Additional tests and procedures	83.47 (386.13)	128.22 (367.03)	44.75 (−5.59 to 95.10)	0.08
Newly prescribed medication	66.78 (253.25)	86.52 (347.46)	19.74 (−20.85 to 60.33)	0.34
Hospitalizations	279.54 (1102.95)	262.77 (1185.29)	−16.77 (−169.73 to 136.18)	0.83

CI, confidence interval; ED, emergency department; GP, general practitioner.

**Table 3:** Healthcare resource use at 1 year

Variable	Pre-hospital rule-out strategy *N* = 434	ED rule-out strategy *N* = 429	*P*-value
One additional ambulance transport *N* (%)	63 (14.7%)	29 (6.8%)	<0.001
Two or more additional ambulance transports *N* (%)	13 (3.0%)	9 (2.1%)	0.40
One ED visit *N* (%)	76 (17.5%)	360 (83.9%)	<0.001
Two or more ED visits *N* (%)	25 (5.8%)	69 (16.1%)	<0.001
One GP consultation *N* (%)	301 (69.4%)	138 (32.2%)	<0.001
Two or more GP consultations *N* (%)	133 (30.6%)	57 (13.3%)	<0.001
One outpatient clinic visit *N* (%)	74 (17.1%)	94 (21.9%)	0.071
Two or more outpatient clinic visits *N* (%)	101 (23.3%)	122 (28.4%)	0.083
CT cardiac *N* (%)	29 (6.7%)	44 (10.3%)	0.059
MRI cardiac *N* (%)	4 (0.9%)	4 (0.9%)	0.99
Echocardiography *N* (%)	93 (21.4%)	94 (21.9%)	0.86
Treadmill *N* (%)	64 (14.7%)	83 (19.3%)	0.072
SPECT *N* (%)	10 (2.3%)	12 (2.8%)	0.65
Other additional tests *N* (%)	137 (31.6%)	135 (31.5%)	0.98
CAG *N* (%)	28 (6.5%)	28 (6.5%)	0.96
PCI *N* (%)	18 (4.1%)	14 (3.3%)	0.49
CABG *N* (%)	1 (0.2%)	1 (0.2%)	0.99
Hospitalization *N* (%)	58 (13.4%)	58 (13.5%)	0.95
Length of hospitalization (days), median (IQR)	0 (0–0)	0 (0–0)	0.99

CABG, coronary artery bypass grafting; CAG, coronary angiography; CT, computed tomography; ED, emergency department; GP, general practitioner; IQR, interquartile range; MRI, magnetic resonance imaging; PCI, percutaneous coronary intervention.

### Quality of life

The EQ-5D-5L questionnaire at 30 days was completed by 331 (76.3%) patients in the pre-hospital rule-out strategy and 311 (72.5%) patients in the ED rule-out strategy. At 30 days, the mean difference in EQ-5D-5L utility score was −0.009 (95% CI −0.048 to 0.030, *P* = 0.65) in the complete cases analysis and −0.011 (95% CI −0.038 to 0.015, *P* = 0.39) in the multiple imputation analysis. The EQ-5D-5L questionnaire at 1 year was completed by 376 (86.6%) patients in the pre-hospital rule-out strategy and 362 (84.4%) patients in the ED rule-out strategy. The mean difference at 1 year in EQ-5D-5L utility score was 0.008 (95% CI −0.033 to 0.050, *P* = 0.69) in the complete cases analysis and 0.005 (95% CI −0.022 to 0.031, *P* = 0.73) in the multiple imputations analysis (*Figure 4*B).

### Cost-effectiveness

The fact that the pre-hospital rule-out strategy resulted in a significant reduction of healthcare costs without significantly affecting QoL implies that the pre-hospital rule-out strategy was the dominant strategy regarding cost-effectiveness. *Figure 4*C shows the CE plane, and *Figure 4*D the CEAC. From the CE plane, it can be inferred that the point estimate (ICER) lies in the lower right-hand quadrant, meaning that the pre-hospital rule-out strategy was dominant by reducing healthcare costs with a slightly better QoL. Consequently, there was no trade-off between healthcare costs and QoL. The CEAC shows that the probability that the pre-hospital rule-out strategy is cost-effective is 100% when the willingness to pay (WTP) for an effect equals zero. When the WTP for an effect increases, more uncertainty in the cost-effectiveness analysis is introduced, and the probability of the pre-hospital rule-out strategy being cost-effective decreases slightly across a range of increasing WTPs but remains well above 80%.

## Discussion

### Principal findings

Our trial shows that pre-hospital rule-out of NSTE-ACS in low-risk patients by a single POC troponin measurement is cost-effective at 1 year, as demonstrated by a sustainable reduction in healthcare costs without affecting QoL. MACE incidence at 1 year was low in the pre-hospital rule-out strategy and the ED rule-out strategy, especially when ACS initially was ruled out, irrespective of whether this was performed at home or in-hospital.

### Safety

Previously, we showed that 30-day MACE incidence in the ARTICA trial was low in patients with low (≤3) pre-hospital HEAR scores and very low in patients for whom an NSTE-ACS was ruled out at initial presentation.^15^ These findings were comparable to previous studies regarding the identification of low-risk patients (HEART score ≤3) at the ED.^12^ Although the ARTICA trial was not powered to estimate the safety of pre-hospital rule-out, long-term follow-up does provide more insight in the safety. One-year incidence of MACE was 4.6% and comparable between both strategies, including MACE at index presentation. In patients with a ruled-out ACS (97% of the study population), the 1-year incidence of MACE was 1.6%, and also comparable between both strategies. Although most studies on HEART score assessment for risk stratification at the ED focused on short-term outcomes, some studies reported long-term outcomes after assessment of a low HEART score. However, these studies were observational, the follow-up duration varied from 3 months to 1 year, and the reported endpoints were heterogeneous.^20–24^ The long-term incidence of MACE in patients with a low HEART score at the ED in the aforementioned studies varied from 0% to 3.1% during follow-up of 3 months to 1 year, which was lower than the 1-year MACE incidence in our study (4.6%). However, an important difference is that patients in the aforementioned studies had a HEART score of ≤3 (including troponin measurement), while patients in our study had a HEAR score of ≤3. As shown in Supplementary material online, * Table S3*, 25 of the 27 patients with ruled-in ACS at index presentation had HEAR scores of 2 or 3 and elevated troponin concentrations because of STEMI or NSTEMI. Adding the troponin component to these HEAR scores would result in HEART scores >3. Therefore, the long-term safety of true low-risk patients is better demonstrated by the ruled-out ACS population instead of the total population. In our study, the 1-year incidence of MACE was 1.7% after ruled-out ACS in the pre-hospital rule-out strategy and 1.4% after ruled-out ACS in the ED rule-out strategy, with a negligible risk difference of 0.2% (95% CI −1.4% to 1.9%, *P* = 0.79). Therefore, in addition to the low 30-day MACE incidence, the final 1-year results of our trial show that the 1-year MACE incidence after the pre-hospital rule-out of NSTE-ACS remains low and is comparable to the incidence after the in-hospital rule-out.^15^ However, in the ruled-out ACS population, 3 patients (0.7%) in the pre-hospital rule-out strategy had an ACS after the first 30 days, in comparison to 0 patients in the ED rule-out strategy. Also, it should be noted that our study was neither formally designed nor powered to investigate the safety of the pre-hospital rule-out strategy. Hence, caution is warranted when interpreting the results of our trial with regards to the safety of the pre-hospital rule-out of NSTE-ACS. The results of our trial do emphasize the importance of a future trial powered to show non-inferiority regarding the safety of pre-hospital rule-out of ACS.

### Cost-effectiveness

The final 1-year results of our trial show that the pre-hospital rule-out of NSTE-ACS was cost-effective, as demonstrated by a sustainable healthcare cost reduction without affecting QoL. The mean healthcare cost difference at 1 year was €717 per patient, which would save an estimated €59M per year if the pre-hospital strategy were to be implemented in all of the Netherlands.^15^ These results are in line with a recent economic evaluation from Australia, which has also shown that pre-hospital risk stratification and POC troponin measurement lead to large long-term healthcare cost reductions.^25^

### Future implications

We believe that the final results of the ARTICA trial indicate that implementation of the pre-hospital rule-out strategy will reduce long-term costs and will help to alleviate the overcrowded ED. The possibility to implement the pre-hospital rule-out strategy depends on multiple factors. First, the level of education of ambulance professionals differs between countries, which might influence the reliability of the HEAR score assessment. However, the overall inter-operator reliability of the HEART score is strong, regardless of the level of education.^26^ Second, ambulance availability differs between countries.^27^ The Netherlands is relatively small and well organized, with short distances and short transportation times. Therefore, in countries with less ambulance availability and longer distances, the amounts of time and money saved by not transporting patients could be even larger. Third, pre-hospital rule-out relies on a well-functioning primary care system. In our trial, after the pre-hospital rule-out of NSTE-ACS, care for the patient was transferred to the GP. In the Netherlands, GPs provide out-of-hours urgent primary care in so-called GP cooperatives, facilitating out-of-hours transfer of the care to a GP.^28^

### Limitations

This study has some limitations. First, follow-up was performed by telephone and e-mail, which could result in a certain degree of misclassification because of the recall bias of the patients.^27^ However, medical records were requested for all hospital visits. Therefore, relevant events and utilized in-hospital healthcare resources were objectively assessed. Second, the EQ-5D-5L questionnaires were not completed by all patients. However, the numbers of missing questionnaires were comparable between groups, and the results before and after multiple imputation were comparable. Third, the POC troponin T assay has a lower limit of detection of 40 ng/L, which is above the 99th percentile of normal on the high-sensitivity troponin T assay in the laboratory (14 ng/L). Therefore, patients with a low POC troponin T concentration (<40 ng/L) could still have mildly elevated troponin concentrations on a high-sensitivity troponin assay. However, the combination of this POC troponin assay and the HEAR score has been shown to identify low-risk patients with low MACE incidence.^13^ Fourth, ambulance paramedics only started the digital case report form (CRF) for patients who they deemed eligible for participation. Therefore, the 1138 patients who were screened for eligibility (as shown in *Figure 1*) do not include patients for whom no CRF was started, and it is unknown how many patients were deemed ineligible without starting the CRF. Fifth, information on clinical outcomes is only known for the patients who signed informed consent and remains unknown for the excluded patients.

## Conclusion

Pre-hospital rule-out of NSTE-ACS in low-risk patients with a single POC troponin measurement was cost-effective, demonstrated by a sustainable healthcare cost reduction at 1 year without affecting QoL. One-year MACE occurrences remained low.

## Supplementary Material

qcae004_Supplemental_File

## Notes

**Conflict of interest.** C.C. reports a grant from ZonMw (The Netherlands Organisation for Health Research and Development) and consulting fees from AstraZeneca. R.J.J.v.K. reports honoraria grants from Bayer and Novartis. P.D. reports grants from Philips, Abbott, and AstraZeneca and consulting fees from Philips. N.v.R. reports grants from Abbott, Philips, Medtronic, and Biotronik and speaker fees from Microport, Abbott, Rainmed, and Bayer. The other authors have no conflicts of interest.

## Data availability statement

Data will be made available one year after publication of the manuscript. Data requests should be submitted to the corresponding author.
