# Supplementary material for: Pre-hospital rule-out of non-ST-segment elevation acute coronary syndrome by a single troponin: final one-year outcomes of the ARTICA randomised trial
Source: Eur Heart J Qual Care Clin Outcomes. 2024 Jan 17;10(5):411–20. doi: 10.1093/ehjqcco/qcae004 (PMC11307197; doi:10.1093/ehjqcco/qcae004)
Supplement: qcae004_Supplemental_File [file qcae004_supplemental_file.docx]

**SUPPLEMENTARY APPENDIX**

Pre-hospital rule-out of non-ST-segment elevation acute coronary syndrome by a single troponin: final one-year outcomes of the ARTICA randomised trial

**Funding**: the trial was funded by the Netherlands Organisation for Health Research and Development (ZonMw ; project number 852001942)

**Trial registration**: The ARTICA trial was registered at Clinicaltrials.gov, number NCT05466591

**Table of contents**

1. Study project team
2. Authorship contributions
3. Data safety and monitoring board (DSMB)
4. Clinical events committee (CEC)
5. List of participating ambulance services and inclusion per region
6. Supplementary table S1: Inclusion- and Exclusion criteria
7. Supplementary figure S1: Modified HEAR score
8. Supplementary table S2: 2018 reference list of the Dutch National Healthcare Institute
9. Supplementary table S3: List of details of the patients with MACE
10. Supplementary table S4: Healthcare costs at one year, within first 30 days and after first 30 days
11. Supplementary table S5: Newly prescribed medications at 12 months
12. **Study project team**

Cyril Camaro (principal investigator), Department of Cardiology, Radboud university medical centre, Nijmegen; Niels v Royen, head department of Cardiology, Radboud university medical centre, Nijmegen ; Goaris W.A. Aarts, department of Cardiology, Radboud university medical centre Nijmegen; Eddy Adang, department of Health Evidence, Radboudumc Nijmegen ; Roland v Kimmenade, Department of Cardiology, Radboud university medical centre ; Marc Gomes, Canisius-Wilhelmina Hospital, department of Cardiology; Eva Ouwendijk, general practitioner; Martijn Rutten, general practitioner IQ Healthcare Radboudumc ; Anouk Hoare, ambulance service Witte Kruis ; Gijs Brok, ambulance service Gelderland-Zuid, Roger v Hout, ambulance service Gelderland-Zuid ; Peter Damman, Department of Cardiology, Radboud university medical centre ; Etienne Cramer, Department of Cardiology, Radboud university medical centre ; Robert-Jan van Geuns, Department of Cardiology, Radboud university medical centre,

1. **Authorship contributions**

CC conceived the idea. GWAA, CC, RJvG, EC, RRJvK, PD and NvR designed the study methodology. EA designed the economical and statistical analyses. CC, GWAA and NvR drafted the manuscript. GWAA, CC, EA and LR designed the statistical analysis plan. The first draft of the manuscript was written by CC, GWAA and NvR. All authors reviewed and revised the final manuscript. All authors agreed with the final version of the manuscript.

1. **Data safety and monitoring board (DSMB) members**

Prof.dr F. Zijlstra, cardiologist, former head department of Cardiology, Erasmus MC Rotterdam, chairman

Prof.dr J. Tijssen, (bio)statistician

Dr E.C.T.H. Tan, trauma surgeon, former chief of Emergency department Radboudumc (2019-2021)

Prof.dr K. Roes, independent statistician

1. **Clinical event committee members**

Prof.dr MJ de Boer, cardiologist

Dr H.A. Bosker, cardiologist

1. **List of participating ambulance services and inclusions per region**


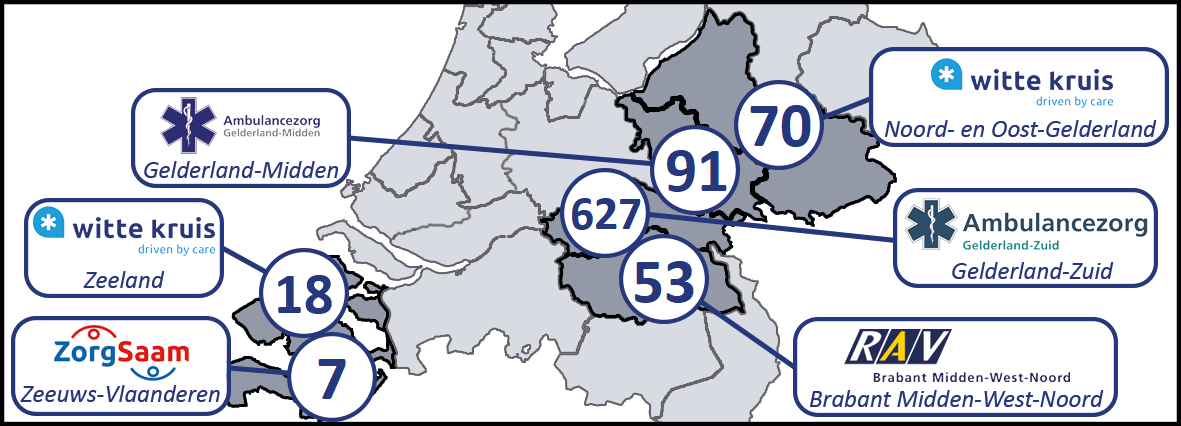


Ambulance service Gelderland-Zuid: 627 patients

Ambulance service Gelderland-Midden: 91 patients

Witte Kruis Noord- en Oost Gelderland: 70 patients

Ambulance service Brabant Midden-West-Noord 53 patients

Witte Kruis Zeeland 18 patients

Zorgsaam Zeeuws -Vlaanderen 7 patients

1. **Supplementary Table S1 - inclusion and exclusion criteria**

| **Inclusion criteria**  ● Age ≥18 years  ● Suspected NSTE-ACS  ● Symptom duration of at least two hours  ● Modified HEAR(T) score ≤3  ● Provided written informed consent  **Exclusion criteria**  ● ST -segment elevation  ● Suspected non-cardiac cause of the symptoms requiring ED visit  ● Comatose state, defined as a GCS score <8  ● Known cognitive impairment  ● Pregnancy  ● Cardiogenic shock (= systolic blood pressure <90mmHg, heart rate >100bpm and peripheral oxygen saturation <90%)  ● Syncope  ● Signs of heart failure  ● Heart rhythm disorders and second-degree or third-degree atrioventricular block  ● Known end-stage renal disease (dialysis and/or MDRD <30mL/min)  ● Suspected aortic dissection or pulmonary embolism  ● Confirmed AMI, PCI or CABG <30 days prior to inclusion  ● Communication issues with the patient and/or language barrier  ● Decision of a present general practitioner to evaluate the patient at the ED  ● Decision of the consultant cardiologist to evaluate the patient at the emergency department  ● Any significant medical or mental condition, which in the investigator’s opinion may interfere with optimal participation in  the study |
| --- |

NSTE-ACS= non ST-segment elevation acute coronary syndrome, ED=emergency department, GCS=Glasgow coma scale, AMI=acute myocardial infarction, PCI=percutaneous coronary intervention, CABG=coronary artery bypass grafting

1. **Supplementary Figure S1 –** modified HEAR score

   **
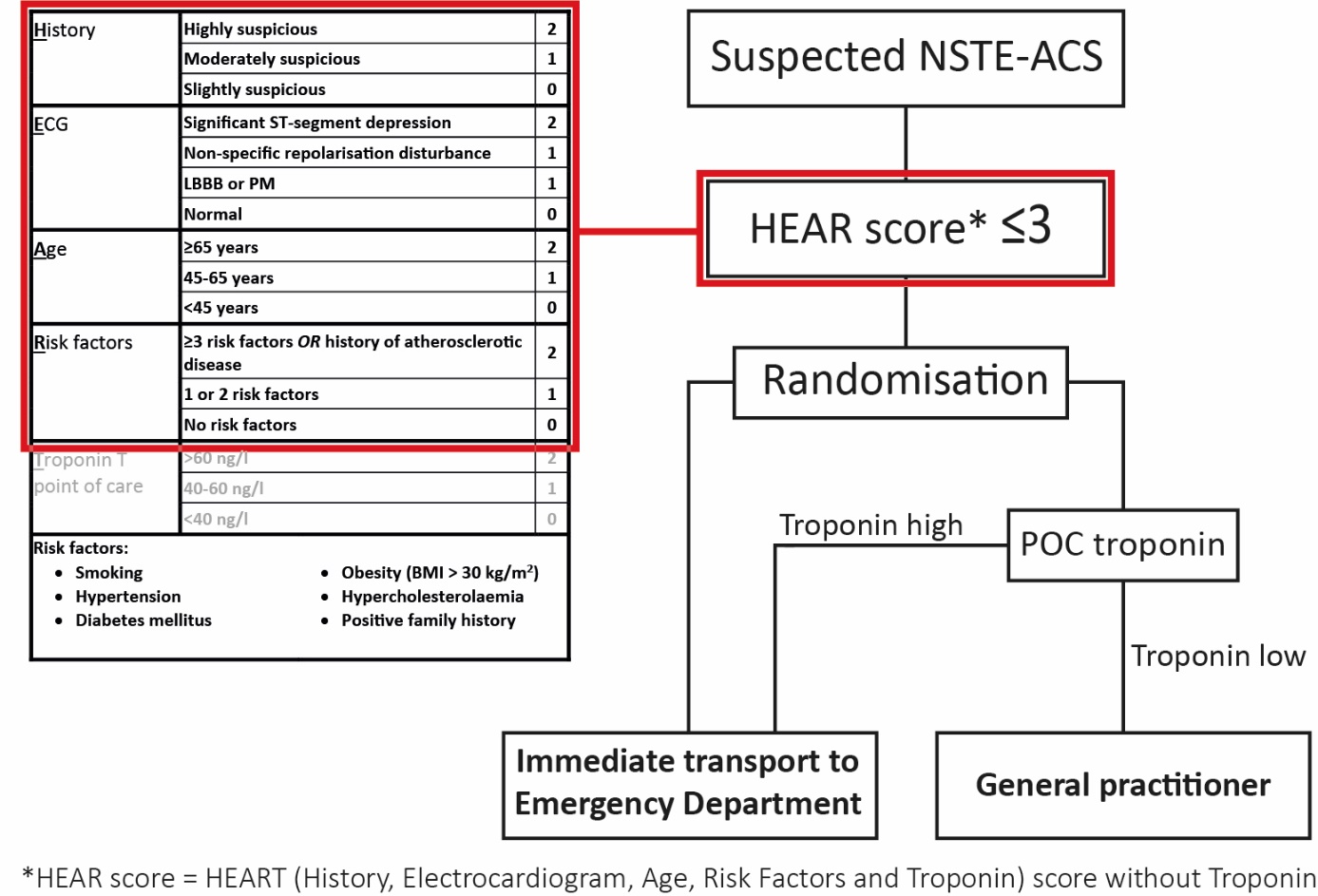
**
2. **Supplementary table S2 -**2018 reference list of the Dutch National Healthcare Institute (EURO)

| **Ambulance** | |
| --- | --- |
| First ambulance visit (inclusion in the trial) | 750 |
| Ambulance transport (first ambulance visit excluded) | 651 |
| **Hospital** | |
| ED: chest pain | 580 |
| ED: other | 275 |
| Outpatient clinic | 97 |
| Ward/CCU per day (weighted average) | 506 |
| **General Practitioner** | |
| GP visit (weighted average) | 33 |
| **Diagnostic tests** | |
| Blood tests at the ED | 53 |
| Second troponin test | 8 |
| ECG | 46 |
| D-dimer | 5 |
| Coronary angiography | 628 |
| Chest X-ray | 47 |
| CT angiography chest | 193 |
| CT cardiac | 301 |
| MRI cardiac | 335 |
| Treadmill | 107 |
| Echocardiography | 82 |
| SPECT scan | 509 |
| **Procedures** | |
| PCI | 4624 |
| CABG | 13438 |

ED=emergency department, CCU=cardiac care unit, GP=general practitioner, ECG=electrocardiogram, CT=computed tomography, MRI=magnetic resonance imaging, SPECT=single photon emission computed tomography, PCI=percutaneous coronary intervention, CABG=coronary artery bypass grafting

1. **Supplementary table S3 –** List of details of the patients with MACE

| **Study number** | **Age** | **Sex** | **HEAR score** | **MACE type (first MACE)** | **Days to MACE** | **Information** |
| --- | --- | --- | --- | --- | --- | --- |
| **Pre-hospital rule-out strategy, ACS ruled-in (n=15)** | | | | | | |
| ARTICA-0184 | 59 | M | 2 | ACS (NSTEMI) | 0 | POC troponin T elevated, direct transport to the ED. Revascularisation performed for NSTEMI. |
| ARTICA-0205 | 58 | F | 3 | ACS (NSTEMI) | 0 | POC troponin T elevated, direct transport to the ED. No obstructive coronary artery disease, diagnosis MINOCA. |
| ARTICA-0261 | 49 | M | 3 | ACS (STEMI) | 0 | POC troponin T elevated, direct transport to the ED. At the ED, the ECG showed ST-segment elevations, diagnosis STEMI, for which revascularization was performed. |
| ARTICA-0316 | 66 | M | 3 | ACS (NSTEMI) | 0 | POC troponin T elevated, direct transport to the ED. Three-vessel disease, for which revascularization was planned (not unplanned) |
| ARTICA-0415 | 49 | M | 3 | ACS (NSTEMI) | 0 | POC troponin T elevated, direct transport to the ED. Revascularisation performed for NSTEMI. |
| ARTICA-0593 | 56 | F | 3 | ACS (STEMI) | 0 | POC troponin T elevated, direct transport to the ED. At the ED, the ECG showed ST-segment elevations, diagnosis STEMI, for which revascularization was performed |
| ARTICA-0665 | 63 | M | 3 | ACS (NSTEMI) | 0 | POC troponin T elevated, direct transport to the ED. Revascularisation performed for NSTEMI. |
| ARTICA-0703 | 41 | M | 3 | ACS (NSTEMI) | 0 | POC troponin T elevated, direct transport to the ED. Revascularisation performed for NSTEMI. |
| ARTICA-0736 | 56 | M | 3 | ACS (NSTEMI) | 0 | POC troponin T elevated, direct transport to the ED. Revascularisation performed for NSTEMI. |
| ARTICA-0772 | 65 | M | 3 | ACS (NSTEMI) | 0 | POC troponin T elevated, direct transport to the ED. Revascularisation performed for NSTEMI. |
| ARTICA-0800 | 51 | M | 2 | ACS (NSTEMI) | 0 | POC troponin T elevated, direct transport to the ED. Revascularisation performed for NSTEMI. |
| ARTICA-0838 | 63 | F | 2 | ACS (NSTEMI) | 0 | POC troponin T elevated, diagnosis NSTEMI, conservative medical treatment |
| ARTICA-0898 | 62 | F | 2 | ACS (STEMI) | 0 | POC troponin T elevated, direct transport to the ED. At the ED, the ECG showed ST-segment elevations, diagnosis STEMI, for which revascularization was performed |
| ARTICA-0927 | 83 | M | 3 | ACS (NSTEMI | 0 | POC troponin T elevated, direct transport to the ED. Three-vessel disease, for which revascularization was planned (not unplanned) |
| ARTICA-0979 | 62 | M | 3 | ACS (NSTEMI) | 0 | POC troponin measurement failed, direct transport to the ED. At ED high-sensitivity troponin elevated, diagnosis NSTEMI. Diffuse three vessel disease, medical treatment. |
| **Pre-hospital rule-out strategy, ACS ruled-out (n=7)** | | | | | | |
| ARTICA-0356 | 55 | F | 3 | ACS (Unstable angina) | 132 | Presentation at the ED for new symptoms, dynamic ECG changes, troponin low. Diagnosis unstable angina for which revascularization was performed. |
| ARTICA-0416 | 92 | F | 3 | Death | 86 | Died from end-stage myelodysplastic syndrome |
| ARTICA-0566 | 48 | M | 3 | ACS (NSTEMI) | 141 | Presentation at the ED for new symptoms, diagnosis NSTEMI for which revascularization was performed. |
| ARTICA-0584 | 43 | M | 3 | ACS (NSTEMI) | 7 | Presentation at the ED for recurrence of symptoms. POC troponin was low at inclusion. At the ED high-sensitivity troponin is very high, diagnosis NSTEMI for which revascularization is performed. |
| ARTICA-0996 | 70 | M | 3 | ACS (STEMI) | 2 | Emergency number called for recurrence of symptoms, ambulance came and obtained an ECG which showed a STEMI. At inclusion, the ECG was normal and POC troponin T was low. Revascularisation was performed for the STEMI. |
| ARTICA-1043 | 59 | F | 3 | ACS (STEMI) | 127 | Presentation at the ED for new symptoms. Diagnosis STEMI, for which revascularisation was performed. |
| ARTICA-1082 | 69 | M | 3 | Death | 140 | The patient was found at home, confused but not unconscious. Ambulance came and while carrying the patient into the vehicle, the patient died. No obduction was performed. |
| **ED rule-out strategy, ACS ruled-in (n=12)** | | | | | | |
| ARTICA-0165 | 62 | M | 3 | ACS (NSTEMI) | 0 | Diagnosis NSTEMI at the ED, for which revascularisation was performed. |
| ARTICA-0306 | 54 | M | 3 | ACS (NSTEMI) | 0 | Diagnosis NSTEMI at the ED, for which revascularisation was performed. |
| ARTICA-0379 | 51 | F | 3 | ACS (NSTEMI) | 0 | Diagnosis NSTEMI at the ED, for which revascularisation was performed. |
| ARTICA-0660 | 60 | M | 3 | ACS (unstable angina) | 0 | Diagnosis unstable angina at the ED, for which revascularisation was performed. |
| ARTICA-0684 | 52 | F | 3 | ACS (NSTEMI | 0 | Diagnosis NSTEMI at the ED, conservative treatment. |
| ARTICA-0752 | 59 | M | 1 | ACS (NSTEMI) | 0 | Diagnosis NSTEMI at the ED, for which revascularisation was performed. |
| ARTICA-0827 | 50 | M | 3 | ACS (NSTEMI) | 0 | Diagnosis NSTEMI at the ED, for which revascularisation was performed. |
| ARTICA-0855 | 63 | M | 3 | ACS (NSTEMI) | 0 | Diagnosis NSTEMI at the ED, for which revascularisation was performed. |
| ARTICA-0882 | 69 | M | 3 | ACS (NSTEMI) | 0 | Diagnosis NSTEMI at the ED, for which revascularisation was performed. |
| ARTICA-0915 | 59 | F | 3 | ACS (NSTEMI) | 0 | Diagnosis NSTEMI at the ED, for which revascularisation was performed. |
| ARTICA-1034 | 49 | M | 3 | ACS (NSTEMI) | 0 | Diagnosis NSTEMI at the ED, no obstructive coronary artery disease. MINOCA. |
| ARTICA-1131 | 42 | M | 2 | ACS (NSTEMI) | 0 | Diagnosis NSTEMI at the ED, for which revascularisation was performed. |
| **ED rule-out strategy, ACS ruled-out (n=6)** | | | | | | |
| ARTICA-0233 | 68 | F | 3 | ACS (unstable angina) | 30 | Admission with symptoms and ECG changes, troponin low. Diagnosis unstable angina, for which revascularisation was performed. |
| ARTICA-0599 | 58 | M | 1 | ACS (unstable angina) | 2 | Admission with symptoms and ECG changes, troponin low. Diagnosis unstable angina, for which revascularisation was performed. |
| ARTICA-0673 | 54 | M | 3 | ACS (unstable angina) | 1 | Admission with symptoms and ECG changes, troponin low. Diagnosis unstable angina, for which revascularisation was performed. |
| ARTICA-0832 | 76 | M | 3 | Death | 41 | The patient died from thyroid cancer. |
| ARTICA-0894 | 47 | F | 1 | Death | 240 | The patient committed suicide. |
| ARTICA-1068 | 52 | M | 1 | Death | 7 | The patient died after an out-of-hospital cardiac arrest. |

1. **Supplementary table S4 –** Healthcare costs at one year, within first 30 days and after first 30 days

|  | **Pre-hospital rule-out strategy  (N=434)** | **ED rule-out strategy**  **(N=429)** | **Mean difference (95% CI)** | **P-value** |
| --- | --- | --- | --- | --- |
| **Healthcare costs** |  |  |  |  |
| Costs at one year (€) | 1931.83 (2784.01) | 2648.63 (2749.56) | 716.80 (347.07 – 1086.51) | <0.001 |
| Costs within first 30 days (€) | 1349.42 (2050.83) | 1960.39 (1807.63) | 610.97 (352.57 – 869.37) | <0.001 |
| Costs after first 30 days (€) | 582.41 (1634.19) | 688.24 (1491.76) | 105.83 (-103.48 to 315.15) | 0.32 |
| **Ambulance transports** |  |  |  |  |
| Costs at one year (€) | 846.00 (291.07) | 831.94 (319.67) | -14.06 (-54.90 to 26.78) | 0.50 |
| Costs within first 30 days (€) | 805.50 (216.40) | 765.17 (98.34) | -40.33 (-62.83 to 17.82) | <0.001 |
| Costs after first 30 days (€) | 40.50 (185.93) | 66.77 (273.40) | 26.27 (-4.94 to 57.48) | 0.10 |
| **GP visits** |  |  |  |  |
| Costs at one year (€) | 52.09 (33.11) | 25.00 (38.55) | -27.09 (-31.89 to -22.29) | <0.001 |
| Costs within first 30 days (€) | 46.19 (26.21) | 17.08 (27.48) | -29.12 (-32.71 to -25.53) | <0.001 |
| Costs after first 30 days (€) | 5.90 (18.04) | 7.93 (25.64) | 2.03 (-0.93 to 4.99) | 0.18 |
| **Hospital visits** |  |  |  |  |
| Costs at one year (€) | 603.94 (1530.36) | 1314.17 (1450.37) | 710.23 (510.97 to 909.48) | <0.001 |
| Costs within first 30 days (€) | 329.15 (1259.02) | 1000.52 (1140.59) | 671.37 (510.81 to 831.94) | <0.001 |
| Costs after first 30 days (€) | 274.79 (788.38) | 313.65 (665.71) | 38.85 (-58.59 to 135.40) | 0.44 |
| **Additional tests and procedures** |  |  |  |  |
| Costs at one year (€) | 83.47 (386.13) | 128.22 (367.03) | 44.75 (-5.59 to 95.10) | 0.08 |
| Costs within first 30 days (€) | 14.42 (56.40) | 22.37 (64.11) | 7.95 (-0.12 to 16.01) | 0.05 |
| Costs after first 30 days (€) | 69.05 (383.26) | 105.86 (353.80) | 36.80 (-12.49 to 86.10) | 0.14 |
| **Medication*** |  |  |  |  |
| Costs at one year (€) | 66.78 (253.25) | 86.52 (347.46) | 19.74 (-20.85 to 60.33) | 0.34 |
| Costs within first 30 days (€) | 3.90 (16.31) | 4.43 (20.73) | 0.52 (-1.97 to 3.01) | 0.68 |
| Costs after first 30 days (€) | 62.88 (239.58) | 82.10 (332.69) | 19.22 (-19.48 to 57.92) | 0.33 |
| **Hospitalisations** |  |  |  |  |
| Costs at one year (€) | 279.54 (1102.95) | 262.77 (1185.29) | -16.77 (-169.73 to 136.18) | 0.83 |
| Costs within first 30 days (€) | 150.26 (821.19) | 150.83 (733.78) | 0.57 (-103.52 to 104.66) | 0.99 |
| Costs after first 30 days (€) | 129.29 (700.98) | 111.94 (714.88) | -17.35 (-111.94 to 77.25) | 0.72 |

CI, confidence interval; ED, emergency department; GP, general practitioner. *Newly prescribed medication only.

1. **Supplementary table S5 –** Newly prescribed medications at 12 months

|  | **Pre-hospital strategy**  **(N= 434)** | **ED strategy**  **(N=429)** | **P-value** |
| --- | --- | --- | --- |
| Proton pump inhibitors | 41 | 83 | <0.001 |
| Betablockers | 42 | 45 | 0.69 |
| Antihypertensives | 36 | 42 | 0.44 |
| Aspirin | 36 | 40 | 0.59 |
| Statins | 32 | 38 | 0.42 |
| Other | 28 | 38 | 0.18 |
| Analgesics | 19 | 25 | 0.33 |
| Anti-inflammatories | 19 | 24 | 0.41 |
| P2Y12-inhibitors | 24 | 16 | 0.21 |
| Nitrates | 19 | 20 | 0.84 |
| Antacida | 6 | 26 | <0.001 |
| Antibiotics | 16 | 12 | 0.46 |
| Heparin & LMWH | 11 | 10 | 0.85 |
| Antiarrhythmics | 10 | 8 | 0.65 |
| Anticoagulants | 7 | 6 | 0.80 |
| Benzodiazepines | 6 | 4 | 0.54 |
